# Metabolic Coordination of Pericyte Phenotypes: Therapeutic Implications

**DOI:** 10.3389/fcell.2020.00077

**Published:** 2020-02-14

**Authors:** Emmanuel Nwadozi, Martina Rudnicki, Tara L. Haas

**Affiliations:** School of Kinesiology and Health Science, Angiogenesis Research Group and Muscle Health Research Centre, York University, Toronto, ON, Canada

**Keywords:** adult stem cell, metabolism, *trans*-differentiation, proliferation, quiescence, fibrosis, regenerative medicine

## Abstract

Pericytes are mural vascular cells found predominantly on the abluminal wall of capillaries, where they contribute to the maintenance of capillary structural integrity and vascular permeability. Generally quiescent cells in the adult, pericyte activation and proliferation occur during both physiological and pathological vascular and tissue remodeling. A considerable body of research indicates that pericytes possess attributes of a multipotent adult stem cell, as they are capable of self-renewal as well as commitment and differentiation into multiple lineages. However, pericytes also display phenotypic heterogeneity and recent studies indicate that lineage potential differs between pericyte subpopulations. While numerous microenvironmental cues and cell signaling pathways are known to regulate pericyte functions, the roles that metabolic pathways play in pericyte quiescence, self-renewal or differentiation have been given limited consideration to date. This review will summarize existing data regarding pericyte metabolism and will discuss the coupling of signal pathways to shifts in metabolic pathway preferences that ultimately regulate pericyte quiescence, self-renewal and *trans*-differentiation. The association between dysregulated metabolic processes and development of pericyte pathologies will be highlighted. Despite ongoing debate regarding pericyte classification and their functional capacity for *trans*-differentiation *in vivo*, pericytes are increasingly exploited as a cell therapy tool to promote tissue healing and regeneration. Ultimately, the efficacy of therapeutic approaches hinges on the capacity to effectively control/optimize the fate of the implanted pericytes. Thus, we will identify knowledge gaps that need to be addressed to more effectively harness the opportunity for therapeutic manipulation of pericytes to control pathological outcomes in tissue remodeling.

## Introduction: What is a Pericyte?

**FIGURE 1:**
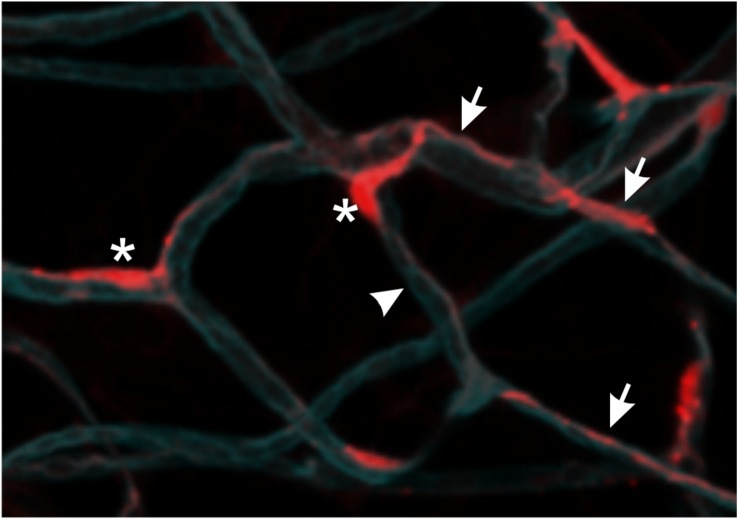
Pericyte morphology. NG2^+^ pericytes are localized to the abluminal surface of adipose tissue capillaries (arrowhead) in mice that express red fluorescent protein (DsRed) under the control of the promoter of chondroitin sulfate proteoglycan 4 (encoding NG2). Pericyte cell bodies (asterisks) are prominently visible due to nuclear localization of DsRed. Thin cytoplasmic processes extend along one or more capillaries (arrows). Capillaries were visualized using Isolectin B4 (cyan).

Pericytes are a heterogeneous population of mural vascular cells that typically reside within the basement membrane on the abluminal surface of most capillaries and some larger blood vessels. The pericyte basement membrane is contiguous with that of the endothelial cells and is composed of extracellular matrix proteins (predominantly collagen IV and the glycoprotein laminin) secreted by both cell types. Morphologically, pericytes are characterized by numerous cytoplasmic processes that emanate from a prominent cell body, tracking along several endothelial cells and occasionally spanning adjacent capillaries (Figure 1). Pericyte protrusions (pegs) insert into endothelial cell invaginations (sockets) at occasional interruptions in the basement membrane, providing structural support as well as direct heterotypic cell–cell communications (Armulik et al., 2005). The current review focuses on the traditionally defined pericytes, but it should be noted that specialized pericytes that deviate from these conventional characteristics reside within various tissues (i.e. stellate cells in liver, mesangial cells in kidneys, reticular cells in bone marrow) (Díaz-Flores et al., 2009).

**FIGURE 2:**
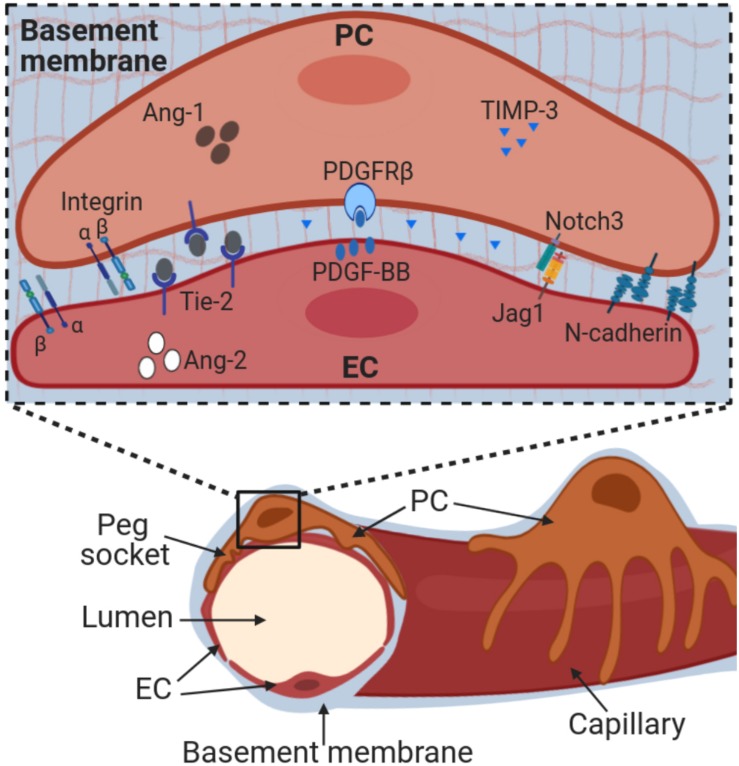
Pericyte-endothelial interactions. Pericytes are embedded into a basement membrane that is shared with endothelial cells and composed primarily by collagen IV and laminins. Adherence to the basement membrane occurs via integrins. Pericyte adherence to endothelial cells at ‘peg and socket’ connections is mediated by N-cadherins and other junctional proteins. Pericyte-endothelial cell cross-talk occurs through multiple molecular pathways, including PDGF-BB/PDGFRβ, Notch receptors and ligands, Angiopoietin-Tie2 and TIMPs. Alteration of these interactions directly impacts pericyte-endothelial interactions and ultimately vessel stability. Ang-1, angiopoietin-1; Ang-2, angiopoietin-2; EC, endothelial cells; Jag1, Jagged1; PC, pericytes; PDGF-BB, platelet derived growth factor; PDGFRβ, PDGF receptor β; TIMP-3, tissue inhibitor of matrix metalloproteinases-3.

During development, pericytes are first attracted to newly formed capillaries via the endothelial-secreted chemoattractant platelet-derived growth factor (PDGF)-BB, which binds to PDGF receptor β (PDGFRβ) on pericytes (Lindahl et al., 1997; Benjamin et al., 1998). Integrin-mediated adhesion of pericytes to laminin helps to maintain expression of PDGFRβ (Durbeej, 2010; Reynolds et al., 2017). Interference with PDGF-BB/PDGFRβ signaling is sufficient to disrupt endothelial-pericyte interactions (Lindahl et al., 1997), indicating that perpetual signaling through PDGFRβ is critical to maintain pericyte localization to capillary endothelial cells. Several other proteins, such as Notch receptors, support the continued close interaction of pericytes with the underlying endothelial cells and play important roles in maintaining pericyte identity (Armulik et al., 2005; Geevarghese and Herman, 2014; Kofler et al., 2015; Ando et al., 2019) (Figure 2). This tight interaction between endothelial cells and pericytes is critical to the stability and maintenance of the integrity of mature capillary networks. Pericytes restrict capillary expansion and promote endothelial cell viability and quiescence through their physical connections and via secretion of paracrine factors (i.e. Ang-1, TIMP-3) (Papapetropoulos et al., 2000; Saunders et al., 2006). Conversely, loss of pericyte contact (following pericyte detachment or apoptosis) reduces endothelial cell survival and promotes capillary regression. Pericytes play additional functions in the vascular compartment, including preservation of capillary barrier function, blood flow regulation, and immunomodulation (Bergers and Song, 2005; Armulik et al., 2011; Geevarghese and Herman, 2014; Navarro et al., 2016). Of note, pericytes also contribute to different cellular processes involved in tissue homeostasis through the potential of differentiating in other cell types (discussed in detail below).

To date, there is no molecular marker known to be unique to pericytes. Thus, a combination of general criteria are commonly used to define pericyte populations, such as perivascular localization, morphology and the expression of one or more recognized molecular markers such as Neural/glial antigen 2 (NG2), PDGFRβ or cluster of differentiation 146 (CD146) (Armulik et al., 2011; Holm et al., 2018). However, these pericyte markers lack specificity. They are expressed to some extent in other cell types (i.e. smooth muscle cells and interstitial cells such as fibro-adipocyte progenitors) and they display variable expression patterns on pericytes across tissues, location within the vascular tree, developmental state and pathological setting (van Dijk et al., 2015; Sacchetti et al., 2016; Kumar et al., 2017).

## Activation, Multipotency and Fate Specificity of Pericytes

Pericytes that reside within an established microvessel network are dominantly quiescent. However, physiological expansion of the capillary network and a variety of pathological conditions trigger their activation and proliferation, which may result in self-renewal or in differentiation. During sprouting angiogenesis, pericytes initially detach from the vessel wall and assist in remodeling the basement membrane to enable endothelial cell sprout formation (Carmeliet and Jain, 2011). Proliferation and migration of pericytes during capillary sprouting ensures pericyte coverage of nascent capillaries. Following sprout formation, pericytes either re-establish pericyte-endothelial cell contacts and return to a quiescent state or they undergo differentiation into smooth muscle cells, resulting in arteriolarization of capillaries (Skalak et al., 1998; Peirce and Skalak, 2003; Volz et al., 2015). Despite the substantial amount of angiogenesis research in past decades, moderately little is known about the molecular pathways that dictate the transition of pericytes between quiescence, proliferation or differentiation.

The differentiation of pericytes into multiple lineages (osteoblasts, chondrocytes and adipocytes) is observed when these cells are cultured (Crisan et al., 2008; Geevarghese and Herman, 2014). This multipotency is analogous to cells belonging to the heterogeneous multipotent stromal population previously referred to as “mesenchymal stem cells” but more commonly described now as “mesenchymal progenitors.” In fact, cultured pericytes display broader multipotency compared to mesenchymal progenitors, including differentiation into vascular smooth muscle cells, myofibroblasts as well as parenchymal cells such as skeletal and cardiac myocytes and neuronal cells (Barron et al., 2016; Ji et al., 2016; Birbrair et al., 2017; Siedlecki et al., 2018; Alarcon-Martinez et al., 2019). The broad multipotency reported for pericytes is the basis on which some researchers postulate that pericytes are the predominant source of tissue resident mesenchymal progenitors (Crisan et al., 2008; da Silva Meirelles et al., 2008). These multipotent characteristics also has led to their growing use for cell therapies to promote tissue healing and regeneration.

Multipotency is not a universal property of all pericytes. For instance, pericytes expressing T-Box transcription factor 18 (Tbx18) failed to display *trans*-differentiation capacity *in vivo* in multiple tissues assessed, including skeletal, cardiac and adipose tissues (Guimarães-Camboa et al., 2017). Notably, not all pericytes express Tbx18 and thus it has been proposed that multipotent pericytes are marked by the absence of Tbx18 (Birbrair et al., 2017; Wörsdörfer and Ergün, 2018). As well, there is evidence that pericyte subsets within and across tissues exhibit distinct transcriptomes and differentiation potentials that may correspond with pre-programed commitment to specific lineages (Birbrair et al., 2013; Sacchetti et al., 2016; Yianni and Sharpe, 2018). This idea is supported by recent single cell profiling of brain and lung derived pericytes^1^ (He et al., 2018; Vanlandewijck et al., 2018) that revealed a non-overlapping expression profile of lineage specific regulators including Runx2 (osteogenesis), Ppar γ (adipogenesis) and Sox-9 (chondrogenesis). Further, single cell sequencing identified sub-populations of adult brain-derived pericytes that exhibited distinct competencies for induced reprograming to a neuronal lineage (Karow et al., 2018).

Effective strategies to modulate pericyte function and *trans*-differentiation are currently lacking. One significant challenge is that pericyte differentiation potential varies uniquely dependent on their tissue/organ of origin (Chen et al., 2015; Pierantozzi et al., 2016; Yianni and Sharpe, 2018). For example, PDGFRβ^+^ZFP423^+^ pericytes within murine adipose tissue readily undergo adipogenesis, thus contributing to adipocyte hyperplasia (Vishvanath et al., 2016). Type-1 and Type-2 pericytes within skeletal muscle, which are classified based on their expression of PDGFRα or Nestin, exhibit exclusive adipogenic or myogenic potential, respectively (Birbrair et al., 2013, 2014). In the brain, pericytes are a potential source of precursors that regenerate neuronal cells (Nakata et al., 2017; Farahani et al., 2019). A recent study revealed a role for epigenetic regulation of pericyte stemness and differentiation potential by demonstrating tissue-specific histone modification patterns within genes that regulate pericyte phenotype, metabolism and fate specificity (Yianni and Sharpe, 2018). Thus, available data support the concept that subsets of pericytes exhibit a degree of pre-programed commitment to specific lineages. Although pericyte differentiation into distinct lineages is achievable *in vitro*, it is still under debate whether pericytes *in vivo* receive the necessary microenvironmental cues, under physiological or pathological conditions, to promote these differentiation events (Wörsdörfer and Ergün, 2018). Overall, mechanisms that regulate the multipotency and tissue-specific pre-programing that contribute to pericyte diversity require further elucidation.

## Metabolic Support of Pericyte Status

**FIGURE 3:**
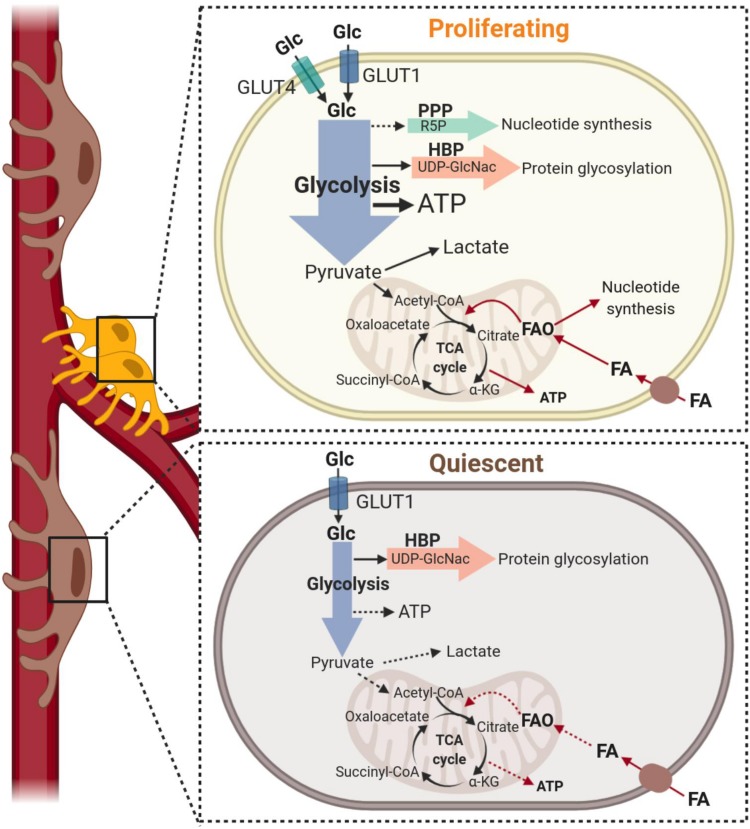
Metabolism of pericytes during proliferation and quiescence. **Top:** Under growth factor stimulation, pericytes increase glucose metabolism (black arrows) to support proliferation. Pericytes increase glucose uptake via GLUT1 and GLUT4 transporters and glucose is metabolized to produce ATP predominantly through glycolysis. This metabolic pathway also provides precursors to protein glycosylation that are essential to the production of capillary basement membrane proteoglycans. Although nucleotide synthesis may be supported by glycolysis via the pentose phosphate pathway (dashed arrow), current data indicate that fatty acid-derived carbons are incorporated into dNTP synthesis in pericytes. Fatty acid oxidation (red arrows) also contributes to the bioenergetic demand during proliferation, generating up to 15% of the total ATP content. **Bottom:** Quiescence is associated with a low metabolic state and downregulation of all metabolic programs. However, the relative contributions of glycolysis, glucose oxidation and fatty acid oxidation (illustrated with dashed arrows) within quiescent pericytes have not been established. α-KG, alpha-Ketoglutarate; ATP, Adenosine triphosphate; FA, fatty acid; FAO, fatty acid oxidation; Glc, glucose; HBP, hexosamine biosynthesis pathway; PPP, pentose phosphate pathway; R5P, Ribose 5-phosphate; TCA, Tricarboxylic acid; UDP-GlcNac, Uridine 5′-diphospho-*N*-acetylglucosamine.

Recent studies have revealed the relevance of metabolic pathways in controlling the acquisition of different phenotypes of vascular and stem cells, indicating promise of novel metabolism-based therapeutic strategies to manipulate the activation status, functions and the fate decisions of native pericytes and those used for regenerative therapies. The contribution of specific metabolic programs to the regulation of cell-state decisions has been investigated extensively in endothelial cells (De Bock et al., 2013; Schoors et al., 2015; Kim et al., 2017; Diebold et al., 2019), whereas the metabolism of pericytes has undergone very limited analysis to date. Below, we will discuss the current knowledge of pericyte metabolism when these cells are instructed to switch from a quiescent to a proliferative state (also illustrated in Figure 3) and in differentiation. Where known, we identify the species and tissue source of pericytes used in each study.

Cells increase the uptake and catabolism of nutrients, particularly glucose, to undergo proliferation. While it was initially believed that the uptake of glucose in pericytes was mediated entirely by the GLUT1 glucose transporter (Mandarino et al., 1994), more recent data demonstrate that transcripts for both GLUT1 and GLUT4 are detectable in human and murine brain pericytes (Castro et al., 2018; He et al., 2018; Vanlandewijck et al., 2018), indicating that glucose uptake can occur via both insulin-dependent and independent pathways. This may explain the greater maximum glucose transport capacity observed in bovine retinal pericytes as compared to endothelial cells (which uptake glucose exclusively through GLUT1) (Mandarino et al., 1994).

Pericyte metabolism has been assessed to the greatest extent in primary cultures of human placental and bovine retinal pericytes (Schoors et al., 2015; Cantelmo et al., 2016). In the proliferative state that exists in cell culture, placental pericytes rely heavily on glycolysis to meet energy demands, with ∼85% of their ATP generation coming from this metabolic pathway (Cantelmo et al., 2016). In fact, only a small decrease in oxygen consumption was observed when proliferating retinal pericytes were treated with oligomycin, an inhibitor of H^+^-ATP-synthase, further illustrating their reliance on glycolytic rather than mitochondrial ATP production (Trudeau et al., 2011). Notably, the inhibition of 6-phosphofructo-2-kinase/fructose-2,6-bisphosphatase 3 (PFKFB3), which impairs glycolysis, was shown to restrain both proliferation and migration of pericytes and to enforce pericyte quiescence *in vivo* and *in vitro* (Cantelmo et al., 2016). This indicates that glycolysis is vital to orchestrate the exit from quiescence in these cells.

Besides providing ATP and reducing cofactors to support anabolic reactions, the catabolism of glucose generates precursors to sustain lipid production, the biogenesis of nucleotides and non-essential amino acids and the synthesis of glycolipids, proteoglycans and substrates for protein glycosylation. Since pericytes have greater maximal rate of glucose transport than endothelial cells, it has been suggested that pericytes channel a greater proportion of glycolytic intermediates into the hexosamine biosynthesis pathway (HBP), which generates *N*-acetylglucosamine for *O*- and *N*-glycosylation of proteins and supports the production of capillary basement membrane proteoglycans (Mandarino et al., 1994). The HBP also directly increases cellular biomass (Wellen et al., 2010) and, therefore, diverting glucose into this side branch of glycolysis may play key roles in pericyte self-renewal by sustaining proliferation and also re-establishing the quiescent state of these cells.

Fatty acid oxidation is estimated to contribute to ∼15% of human placental pericyte ATP production (Cantelmo et al., 2016). Pericytes express CD36 and several fatty acid transporter proteins (FATPs) (Winkler et al., 2014), but the relative contribution of these transporters or others in fatty acid uptake remain to be defined. The cultured placental pericytes use fatty acid-derived carbons for the synthesis of deoxyribonucleotides, which implies that fatty acid oxidation supports the proliferation of pericytes (Schoors et al., 2015). While fatty acid oxidation is the predominant metabolic pathway in quiescent endothelial cells (Kalucka et al., 2018), its contribution to the metabolic activity of quiescent pericytes remains to be established. Moreover, the relevance of glutamine metabolism in supporting the proliferative or quiescent states of pericytes is also unknown, although this generally constitutes a major nutrient that is consumed by proliferating cells.

It is well-recognized that the differentiation of multipotent cells is accompanied by shifts in cellular metabolism. Glycolysis is associated with stem cell pluripotency (Folmes et al., 2013; Gu et al., 2016) as it fuels cytosolic acetyl-CoA synthesis, which is essential to maintain histone acetylation required for a multipotent epigenetic state (Moussaieff et al., 2015). Furthermore, glycolysis limits cellular reliance on oxygen and the generation of reactive oxygen species (ROS) (Chen et al., 2008; Suda et al., 2011), which in turn play a critical role in the differentiation of stem cell into various cell populations (adipocytes, osteocytes, chondrocytes, myocytes) (Boopathy et al., 2013; Higuchi et al., 2013; Mateos et al., 2013). Accordingly, stem cell differentiation is usually associated with upregulation of mitochondrial capacity and a substantially higher use of OXPHOS (Funes et al., 2007; Zhang J. et al., 2012; Coller, 2019), which leads to increased levels of ROS. Thus, modulating the glycolytic metabolism of pericytes may not only influence the switch from a quiescent to proliferative state but may also be centrally involved in maintaining the stemness of pericytes. However, no study to date has elucidated the metabolic alterations that accompany the cell-type-specific fate decisions in pericytes.

## Molecular Pathways Coordinating Pericyte State and Metabolism

**FIGURE 4:**
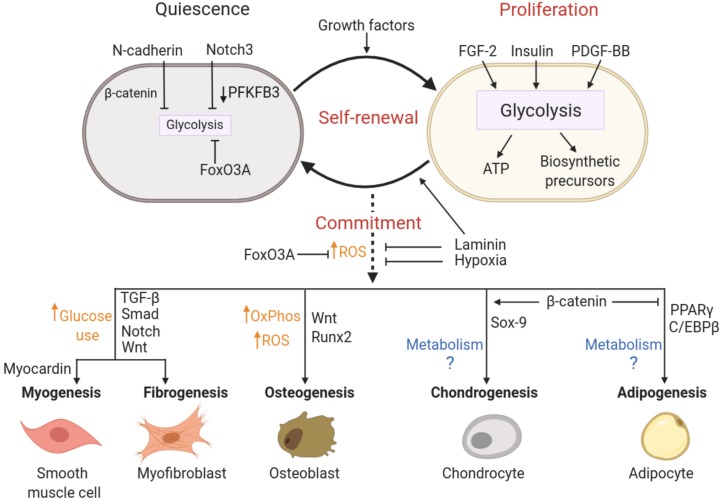
Putative molecular pathways coordinating pericyte state and metabolism. Activation, multipotency and fate specificity of pericytes are governed by different molecular pathways that may involve coordinated metabolic shifts. N-cadherin, Notch3 and FoxO3A signaling are associated with the maintenance of a quiescent state in pericytes by limiting cellular metabolism (mainly glycolysis), whereas pericyte proliferation is stimulated by growth factors and supported by a concomitant increase in glycolytic activity. Self-renewal is achieved by cycles of proliferation followed by the return to a quiescent state, which may be promoted by cell adhesion to laminin. Commitment and *trans*-differentiation may be triggered by an increase in oxidative stress, while unique lineage specifications are determined by a combination of distinct lineage drivers and metabolic reprograming. Conversely, FoxO3A signaling, antioxidant agents, laminin and hypoxia can repress commitment and *trans*-differentiation events. See text for details on the relevant studies. ATP, Adenosine triphosphate; FGF-2, Fibroblast growth factor; FoxO3A, Forkhead BoxO3A; OxPhos, oxidative phosphorylation; PDGF-BB, platelet derived growth factor; PFKFB3, 6-phosphofructo-2-kinase/fructose-2,6-biphosphatase 3; ROS, reactive oxygen species.

The metabolic programing that supports pericyte status is under the control of signal transduction pathways that in turn are governed by local levels of stabilizing and mitogenic factors. Below, we will discuss molecular mechanisms that enforce quiescence, favor proliferation or induce commitment and differentiation of pericytes by driving changes in cellular metabolism. These pathways are summarized in Figure 4.

### Quiescence

The quiescent state of pericytes is preserved by cell-intrinsic programs but also by cell-cell interactions. This is achieved in large part by the cell adhesion protein N-cadherin, which mediates heterotypic cell contacts between endothelial cells and pericytes (Gerhardt et al., 2000). Human placental pericyte glycolytic activity is inversely associated with the expression level of N-cadherin (Cantelmo et al., 2016). It has been shown that N-cadherin sequesters β-catenin to the plasma membrane, which may lower β-catenin-dependent promotion of proliferation as was shown to occur in tumor cells (Nadanaka et al., 2018). This effect may be mediated through the regulation of metabolic pathways since c-Myc, a major target gene of the β-catenin/TCF transcription factor complex, is a well-established effector of glycolysis (Li and Wang, 2008). Metabolic state of the cell also influences N-cadherin expression in tumor vessel pericytes, as glycolytic inhibition using the PFKFB3 inhibitor increased N-cadherin protein level and enhanced pericyte adhesion to endothelial cells (Cantelmo et al., 2016).

Besides the regulation imposed by cell adhesion molecules, pericyte quiescence is promoted by Notch signaling. Notch3 on pericytes interacts directly with Jagged-1 on endothelial cells to promote pericyte-endothelial cell adhesion while also inhibiting pericyte proliferation and migration (Liu et al., 2009, 2010; Schulz et al., 2015; Ji et al., 2016). Notch signaling is implicated in the regulation of quiescence and metabolism in many cell types (Iso et al., 2003; Liu et al., 2010; Koch et al., 2013), through diverse actions that include downregulated expression of glycolytic enzymes such as PFKFB3 (De Bock et al., 2013; Bayin et al., 2017). Thus, pericyte quiescence enforced by interactions with endothelial cells via Notch may involve metabolic reprograming driven by this signal pathway.

A similar enforcement of quiescence may be promoted by the Forkhead box O (FOXO) transcription factors. This family of transcriptional regulators maintains cellular quiescence in adult stem cells through repression of metabolic pathway genes and cell cycle activators (Liang and Ghaffari, 2018). FOXO3A was shown to be the predominant FOXO family member produced by pericytes (Teichert et al., 2017). In muscle satellite cells, FOXO3A promotes quiescence, in part through enhancing the expression of Notch1 and 3 (Gopinath et al., 2014; Yue et al., 2017), suggesting a similar function may occur in pericytes. However, elevated levels of nuclear FOXO3A in pericytes (due to Ang2-dependent decrease in Tie-2 activation) was associated with a phenotype shift in pericytes that promoted migration and overall, increased capillary growth (Teichert et al., 2017). Thus, although the function of FOXO proteins in governing quiescence via suppression of metabolic activity has been well-established in many other cell types including endothelial cells, these functions require further investigation in pericytes.

### Proliferation

Growth factors that stimulate pericyte proliferation increase the uptake and utilization of available nutrients. For example, insulin promotes glucose uptake and induces glucose-mediated proliferation of bovine retinal pericytes (King et al., 1983). PDGF-BB is another major growth factor for pericytes. Excessive PDGFR activation is responsible for driving the proliferation of pericytes in kidney disease (Chen et al., 2011). Since this growth factor is a strong driver of glycolysis in cancer cells and smooth muscle cells (including increased expression of glucose transporters) (Wang et al., 1999; Ran et al., 2013; Xiao et al., 2017) it is very likely that PDGFRβ signaling enhances glycolysis to meet the energy and biomass demands of proliferating pericytes. Fibroblast growth factor (FGF)-2 also exerts mitogenic influences on pulmonary artery-associated pericytes and can provoke excessive pericyte proliferation in pathological contexts (Ricard et al., 2014). These effects may be mediated by metabolic reprograming, as it has been shown that FGF-2 can coordinate endothelial cell proliferation and migration by increasing the glycolytic capacity of these cells (Yu et al., 2017).

### Commitment and Differentiation

Generally, “stemness’ of pericytes is retained through cycles of proliferation. Maintenance of the stemness and multipotent state of pericytes is highly dependent on interactions with the basement membrane protein laminin. In the absence of this protein, brain pericytes develop the properties of a contractile cell (Yao et al., 2014). Stemness of pericytes is also tuned by other microenvironmental cues and transcription factors that are established regulators of genes encoding metabolic pathway components. Thus, changes in metabolism are expected to accompany and to facilitate differentiation of pericytes.

For example, it has been reported that hypoxia represses the differentiation of CD146^+^ human umbilical cord perivascular cells (presumptive pericytes), allowing them to proliferate and maintain their multipotency (Tsang et al., 2013). Since hypoxia upregulates glycolytic activity, these observations imply that a metabolic switch to oxidative metabolism could provoke lineage commitment of pericytes. Accordingly, signaling pathways involved in the regulation of antioxidant defenses, as well as antioxidant compounds, may directly impact the differentiation of pericytes. For example, besides regulating metabolic pathways, FOXO proteins protect cells against oxidative stress via transcriptional upregulation of antioxidant defense proteins (superoxide dismutase, catalase) (Carter and Brunet, 2007). Depletion of FOXO3A results in limited self-renewal and greater commitment of neural stem cells, hematopoietic stem cells, satellite cells (Miyamoto et al., 2007; Tothova et al., 2007; Paik et al., 2009; Gopinath et al., 2014). Antioxidant treatment was shown to improve self-renewal in FoxO3^–/–^ hematopoietic stem cells and in neural precursors, highlighting a critical role for the regulation of oxidant stress in determining commitment (Tothova et al., 2007; Paik et al., 2009). However, the implementation of metabolic switching is lineage-specific (Cliff et al., 2017) and cannot be generalized across all stem cell populations. Importantly, fate decisions also rely on the concomitant upregulation of specific lineage drivers that guide cell-type specific transcriptional programs. Some knowledge of these signal pathways has been established for pericyte differentiation, which we summarize below for three of the most-studied pericyte fates.

### Myofibroblast/Smooth Muscle Cell Differentiation

The transcriptional regulator myocardin is a major lineage driver for smooth muscle cell differentiation (Li et al., 2003; Long et al., 2008; Alexander and Owens, 2012). In turn, TGF-β -Smad/Notch signaling augment pericyte differentiation into smooth muscle cells (Meyrick et al., 1981; Chambers et al., 2003; Alexander and Owens, 2012; Volz et al., 2015). Similarly, the TGFβ, Notch and Wnt signaling pathways contribute to pericyte conversion to myofibroblasts (Kirton et al., 2006; Cheng et al., 2008; Andersson-Sjöland et al., 2016; Aimaiti et al., 2019). Wnt signaling is robustly activated in lung and kidney pericytes following tissue injury and correlates temporally with the onset of fibrosis (Kirton et al., 2007; Andersson-Sjöland et al., 2016). Given that TGF-β-dependent differentiation of fibroblasts to myofibroblasts was associated with increased glucose uptake and glutaminolysis (Bernard et al., 2015, 2018; Andrianifahanana et al., 2016), it is tempting to speculate that this also applies to the conversion of pericytes to myofibroblasts, although this remains to be demonstrated experimentally. In addition, epigenetic modulation of histone marks may ‘lock in’ myofibroblast differentiation, as seen in pericytes that exhibited both repressive marks on PPARγ and activating marks on fibrotic genes (Mann et al., 2010; Perugorria et al., 2012; Zeybel et al., 2017).

### Osteo/Chondrogenic Differentiation

Expression of the transcription factor Runx2 (Osf2/Cbf1a) is a hallmark of pericytes that are poised to undergo osteogenic or chondrogenic differentiation (Doherty and Canfield, 1999; Farrington-Rock et al., 2004). Osteo-inductive factors such as bone morphogenetic proteins (BMP) increase Runx2, which then interacts with osteoblast-specific *cis*-acting elements to promote the expression of osteogenic genes (Ducy et al., 1997; Zebboudj et al., 2003; Gao et al., 2013). During chondrogenesis, Runx2 is downregulated while Sox-9, the master regulator of chondrogenesis, is upregulated (Bi et al., 1999; Akiyama et al., 2002). Interplay between Sox-9 and β-catenin drives chondrogenesis while suppressing other lineages, such as adipogenesis (Akiyama et al., 2004; Kirton et al., 2007).

Metabolic state may contribute to determining pericyte fate decision toward osteogenesis. Runx2 expression itself is glucose-dependent (Wei et al., 2015) and its gene regulatory influences favor glycolysis over oxidative respiration (Choe et al., 2015). However, osteogenesis in mesenchymal cells involves a progressive switch toward oxidative metabolism, which is necessary for complete differentiation (Chen et al., 2019). In line with this, pericyte osteogenic differentiation was markedly reduced under hypoxic conditions, suggesting a reliance on oxidative phosphorylation and/or oxidant stress to elicit complete osteogenic differentiation (Byon et al., 2008; Tsang et al., 2013). FOXO1, which represses glycolysis, plays a significant role in restraining osteoblast differentiation. It does this through lowering oxidant stress as well as by interfering with Wnt-dependent transcriptional programs (Dowell et al., 2003; Rached et al., 2010; Chen et al., 2019). The influence of FOXO transcriptional regulators in pericyte lineage differentiation remains to be established.

### Adipogenesis

The peroxisome proliferator activated receptor gamma (PPARγ) transcription factor is recognized as a master regulator of pre-adipocyte differentiation and metabolism and its expression is strongly stimulated by exposing cultured pericytes to adipogenic factors (Farrington-Rock et al., 2004; Lefterova et al., 2014). C/EBPβ is proposed to play a key transcriptional priming role through binding to closed chromatin ‘hotspots’ with which PPARγ subsequently interacts (Lefterova et al., 2014). The majority of pericytes in lung and brain express C/EBPβ, while only a small percentage of these cells express PPARγ (He et al., 2018; Vanlandewijck et al., 2018), which appears consistent with the concept that a portion of quiescent pericytes are poised to respond to adipogenic cues. In mouse skeletal muscle, Type-1 (PDGFRα^+^) pericytes exhibit adipogenic potential and were shown to contribute to the generation of adipocytes when transplanted into glycerol-injured muscles (Birbrair et al., 2013). Further, pericytes in human and murine skeletal muscle were reported to express leptin mRNA, the levels of which increased under obesogenic conditions (Nwadozi et al., 2019). PPARγ also may be expressed in activated but non-adipogenic pericytes (i.e. proliferating pericytes *in vitro*) and several studies point to additional roles of PPARγ beyond driving adipogenesis. In hematopoietic stem cells, PPARγ suppresses self-renewal while promoting cell differentiation, in large part through strong suppression of glycolysis (Guo et al., 2018). However, hepatic stellate cells (specialized pericytes within the liver) rely on PPARγ signaling to promote fatty acid oxidation, which serves to both maintain their quiescence and prevent myofibroblast differentiation (She et al., 2005; Zhu et al., 2010).

## Effects of Nutrient Excess on Pericyte Metabolism and Differentiation

### Survival and Activation

**FIGURE 5:**
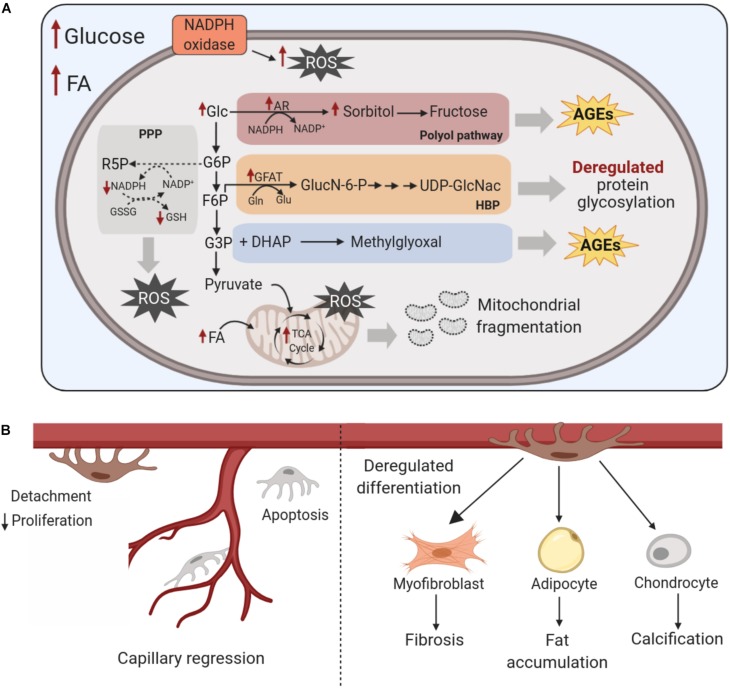
Effects of chronic nutrient excess on pericyte metabolism and *trans-*differentiation. **(A)** Chronic exposure to high glucose levels of pericytes disrupts glucose metabolism and increases diversion of glycolytic intermediates into the polyol and hexosamine biosynthesis pathways, elevates NAD(P)H oxidase activity and increases cellular oxidative stress; This gives rise to augmented ROS levels with the exhaustion of antioxidant defenses, the formation of harmful AGEs, enhanced glycosylation of proteins and fragmentation of mitochondria. Increased fatty acid levels also exacerbate cellular oxidative stress, with concomitant disruptions to metabolic pathways including mitochondrial function. **(B)** These disruptions in the metabolism of pericytes increase their detachment from the vessel wall, lower their proliferation and increase apoptosis, ultimately leading to capillary regression. These pathological metabolic shifts also impact pericyte multipotency by promoting their *trans*-differentiation to myofibroblasts, adipocytes and chondrocytes, which contribute to interstitial fibrosis, fat accumulation and vessel calcification, respectively. AR, aldose reductase; AGEs, advanced glycation end products; DHAP, dihydroxyacetone phosphate; F6P, fructose-6-phosphate; FA, fatty acids; G3P, glyceraldehyde-3-phosphate; G6P, glucose-6-phosphate; GFAT, glutamine fructose-6-phosphate amidotransferase; Glc, glucose; GSSG, glutathione disulfide; GSH, glutathione; HBP, hexosamine biosynthesis pathway; NADPH, nicotinamide adenine dinucleotide phosphate; ROS, reactive oxygen species, TCA, Tricarboxylic acid; UDP-GlcNAc, uridine diphosphate *N*-acetylglucosamine.

Nutrient availability can impact pericyte state in several ways. Hyperglycemia acutely increases glucose metabolism (Giacco and Brownlee, 2010). In the short term, increased levels of glucose enhance glycolysis and glucose oxidation, thus supporting the activation of cellular processes associated with proliferation and migration in cultured rat retinal pericytes (Shah et al., 2013). However, glucose oxidation increases ROS production. In turn, ROS-mediated inhibition of glycolytic enzymes (i.e. GAPDH, PFK) causes the glycolytic intermediates to stall and divert through side-branches of glycolysis (PPP, hexosamine, polyol), thus increasing synthesis of advanced glycation end-products (AGE) (Giacco and Brownlee, 2010; Tang et al., 2012; Mullarky and Cantley, 2015). Increased flux through the polyol pathway consumes NADPH in the process of aldose reductase generation of sorbitol. The continual depletion of NADPH may impair the regeneration of reduced glutathione (GSH), which lowers overall cellular oxidant buffering capacity. High glucose-induced oxidative stress also is sustained by elevated cytosolic NAD(P)H oxidase activity (Giacco and Brownlee, 2010; Tang et al., 2012). These disruptions are illustrated in Figure 5. In pericytes, the associated increase in cellular ROS that accompanies these metabolic disturbances leads to decreased proliferation, fragmentation of mitochondria (with an associated decline in oxygen consumption) and increased apoptosis (Manea et al., 2004, 2005; Mustapha et al., 2010; Trudeau et al., 2011). In rodent retinal and cerebral cortex pericytes, increased aldose reductase-dependent production of sorbitol (Kennedy et al., 1983; Berrone et al., 2006) enhances cellular osmotic stress and endoplasmic reticulum- stress. This may contribute to increased pericyte apoptosis, as was documented in porcine retina and rat lens explants (Takamura et al., 2008; Zhang P. et al., 2012). In fact, NADPH oxidase production of ROS, rather than mitochondria-derived ROS, was found to be instrumental in driving glucose-induced apoptosis of retinal pericytes (Mustapha et al., 2010).

Hyperglycemia also elevates flux through the hexosamine pathway in pericytes by increasing production of the rate-limiting enzyme glutamine:fructose-6-phosphate aminotransferase (GFAT) (Semba et al., 2014). The resultant increase in *O*-GlcNac-modification of proteins, including insulin receptor and PDGFRβ, interferes with mouse retinal pericyte migration (Gurel et al., 2013). FOXO1 may exert a pro-apoptotic function in pericytes in diabetic retinopathy based on the observation that cultured bovine retinal pericytes stimulated with TNFα or AGE products exhibited FoxO1-dependent apoptosis and that FOXO1 RNA interference lowered retinal pericyte apoptosis in diabetic rats (Behl et al., 2009; Alikhani et al., 2010). This suggests that increased conversion of glycolytic intermediary glyceraldehyde 3 phosphate to AGEs can influence a transcription factor that itself is a cornerstone in the regulation of cell survival, metabolism and proliferation/differentiation. Within the retina, prolonged hyperglycemia ultimately is associated with pericyte migration away from the capillary or pericyte ‘dropout,’ which decreases pericyte coverage of the capillary structure, compromising capillary integrity and provoking capillary regression (Pfister et al., 2008; Corliss et al., 2019; Hayes, 2019).

Prolonged exposure to increased levels of fatty acids causes insulin resistance and lipid toxicity in many cell types (Yazıcı and Sezer, 2017). To date, there has been limited investigation of these effects in pericytes. One report indicated that exposure of cultured bovine retinal pericytes to high doses of the fatty acid palmitate increased NAD(P)H oxidase–mediated oxidative stress, with concomitant disruptions to metabolic pathways including mitochondrial function (Cacicedo et al., 2005). Palmitate treatment of cultured retinal pericytes also appears to exacerbate the anti-proliferative, pro-apoptotic effects of AGEs on pericytes, indicating additive effects of these metabolic perturbations (Yamagishi et al., 2002; Cacicedo et al., 2005; Ding et al., 2014). These studies imply that high fatty acids will contribute to oxidative stress-induced damage to pericytes.

### Differentiation Potential

Exposure to excess glucose influence pericyte multipotency in several ways. Neural pericytes cultured in high glucose have increased production of AGEs and up-regulated expression of pro-fibrotic TGF-β, favoring myofibroblast differentiation (Shimizu et al., 2011). Notably, hyperglycemia also increases core fucosylation of the TGF receptors-type I and II (TGFβRI and TGFβRII), which enhances its activation of downstream signals, resulting in hypersensitivity to TGFβ ligands (Sun et al., 2017; Wang et al., 2017). This enhanced core fucosylation of TGFβRI is a key determinant of mesangial pericyte myofibroblast *trans*-differentiation and interstitial fibrosis in mouse models of kidney disease (Lin et al., 2011; Shen et al., 2013; Wang et al., 2017). Diabetes also increases the propensity of muscle- and bone-marrow-derived pericytes to differentiate into adipocytes *in vitro*, while limiting their capacity to promote myogenesis or angiogenesis (Vono et al., 2016; Ferland-McCollough et al., 2018; Mangialardi et al., 2019). These effects were proposed to be linked to elevated ROS. Increased cellular ROS (via NADPH oxidase) also contributes to myofibroblast and osteogenic differentiation in smooth muscle cells and pericytes in diabetic vessels (Byon et al., 2008; Barnes and Gorin, 2011; Raaz et al., 2015). Fatty acid exposure may provoke a similar transition of mesangial pericytes to myofibroblasts by increasing TGF-β secretion (Mishra and Simonson, 2008). In sum, these studies indicate the potential for nutrient imbalances that disturb intracellular metabolism and elevate oxidative stress to trigger inappropriate lineage commitment and *trans*-differentiation of pericytes.

## Clinical Relevance

### Correcting the Functions of Native Pericytes

Establishing the molecular and metabolic switches that control pericyte behavior has the potential to direct the development of new therapeutic opportunities for diseases involving pericyte dysfunction. Targeting pathways that regulate pericyte survival/apoptosis are under exploration as potential therapies in diabetic retinopathy. As previously described, the accumulation of ROS and AGEs induces pericyte apoptosis, ultimately contributing to the capillary loss and fluid leakage that occur in diabetic vasculopathies (Armulik et al., 2011; Ferland-McCollough et al., 2017; Hayes, 2019). Thus, identification of enzyme modifiers that effectively limit glucose uptake or divert the flux of glucose away from “damage-inducing” metabolic pathways (i.e. polyol; AGE-producing) may protect pericytes from high glucose-induced damage and apoptosis by minimizing ROS and AGE accumulation.

On the opposite spectrum, excessive pericyte proliferation promotes disease progression in pulmonary artery hypertension (PAH), hypoxia-induced retinopathies (characterized by excessive neovascularization), and kidney and liver fibrosis (Rabinovitch, 2012; Ferland-McCollough et al., 2017; Dubrac et al., 2018). Studies using pericytes derived from PAH patients indicate that aberrant pericyte proliferation and migration is fueled by glycolysis, as these cells had elevated glycolytic capacity and lower mitochondrial activity (Rabinovitch, 2012; Yuan et al., 2016). Thus, repression of pericyte glycolytic activity could provide a useful therapeutic approach in this disease. Recently, siRNA-mediated knock-down of PDK4 (which suppresses mitochondrial activity in favor of glycolysis by inhibition of pyruvate dehydrogenase) was shown to increase PAH-derived pericyte mitochondrial function, lower their proliferation and improve their interactions with endothelial cells (Yuan et al., 2016), demonstrating promise of this conceptual approach. Similar strategies may be useful in regulating pericyte proliferation in chronic kidney disease or retinopathies, although these approaches remain to be tested.

Excessive differentiation of pericytes into smooth muscle cells also occurs in PAH and contributes to the pathogenesis and severity of the disease (Rabinovitch, 2012; Ricard et al., 2014). In general, the pathological differentiation of pericytes into myofibroblasts is a hallmark of fibrotic kidney and liver diseases (Humphreys, 2018; Parola and Pinzani, 2019). Notably, lineage tracing also demonstrated that *trans*-differentiation of PDGFRβ+ pericytes into fibroblasts occurs within a tumor environment, which causes alterations to capillary structure and interstitial matrix components that ultimately enhance tumor invasion and metastatic potential (Hosaka et al., 2016). *In vivo* pathological activation of osteogenesis/chondrogenesis within pericytes can lead to ectopic ossification (Farrington-Rock et al., 2004; Kirton et al., 2007). This also contributes to large vessel calcification in atherosclerosis (Canfield et al., 2000; Tintut et al., 2003). While efforts to date have focused almost exclusively on disrupting the initiation of ligand-activated receptor signaling (i.e. disproportionate TGF-β or PDGF-BB signaling that provokes smooth muscle or myofibroblast differentiation in these diseases) (Humphreys et al., 2010; Duffield et al., 2013; Hung et al., 2013; Hosaka et al., 2016), it is plausible that coordinated manipulation of metabolic pathways has the potential to achieve more effective repression of these pathological forms of pericyte differentiation. However, more discrete definition of the metabolic switches associated with specific lineage differentiation will be required to take advantage of these opportunities.

### Improving the Use of Pericytes as a Tool for Regenerative Medicine

The capacity to fine-tune pericyte metabolism may provide mechanisms to optimize the function of pericytes used in regenerative medicine, through enhancing the survival and participation of these cells despite the unfavorable conditions found within the host ischemic or injured tissues. Pericytes, and related cell populations that have been referred to as ‘pericyte-like adventitial cells,’ ‘fibro-adipocyte progenitors’ and ‘mesenchymal stromal cells’ have been tested in various types of cell-based therapies and as tools for tissue bioengineering (Zamora et al., 2013; König et al., 2016; Avolio et al., 2017; Murray et al., 2017). To date, pericyte injections have been utilized in pre-clinical studies to promote muscle repair, recovery from muscle or cardiac ischemia, bone/skin injury, diabetic retinopathy (including the clinical trial RETICELL) (Dar et al., 2012; Mendel et al., 2013; Siqueira et al., 2015; Thomas et al., 2017; Alvino et al., 2018; Munroe et al., 2019). The expectation is that pericyte delivery will improve outcomes by one or more actions: secretion of factors that support functions of native cells; replacement of existing ‘damaged’ or ‘dead’ pericytes; differentiation into functional vascular or muscle cells (Caplan, 2009; Cathery et al., 2018).

The value of pericytes as a tool for cellular therapy is highly dependent on their capacity to survive and function within the environment to which they are delivered. The relative reliance of proliferating pericytes on glycolytic vs. oxidative metabolism may help to support pericyte survival when they are injected into ischemic cardiac or skeletal muscle (Cathery et al., 2018). However, conditions within the host tissue microenvironment (i.e. oxidative stress, hyperglycemia, excess TGF-β or PDGF-BB) may impede appropriate activation, proliferation, differentiation and function of healthy donor pericytes, as has been demonstrated within the ischemic limbs of db/db mice (Hayes et al., 2018). Further, the pericyte secretome will vary between pro and anti-inflammatory dependent on the local environment/stimulus (Chen et al., 2013; Gaceb et al., 2018). For example, inflammatory stimuli and AGE will enhance secretion of pro-fibrotic TGF-β, and this could influence the extent to which transplanted pericytes would evoke a fibrotic response in the host tissue.

Moreover, the success of pericyte-based therapies may be limited by the patient’s pre-existing disease. Although autologous transplantation is preferable to use, these cells may be deficient in number and/or function (Fadini et al., 2017, 2019; Teng and Huang, 2019). For example, diabetic individuals have fewer skeletal muscle and bone-marrow derived pericytes than age-matched non-diabetics (Tilton et al., 1981; Mangialardi et al., 2019). Additionally, the ‘stemness’ of these cells may be compromised. Muscle-derived pericytes from diabetic patients exhibited greater adipogenic potential and reduced capacity to promote angiogenesis, which was related to enhanced oxidative stress-dependent damage (Vono et al., 2016). Similarly, bone marrow-derived pericytes from type 2 diabetic patients displayed less Akt activation, which associated with lower survival and proliferation (Mangialardi et al., 2019). Ideally, manipulation of these cells *in vitro* could be used to correct these deficiencies and to condition the cells for more effective functions once transplanted.

## Current Challenges and Future Directions

An ongoing challenge in the field of pericyte biology is the inherent heterogeneity of this cell population. This has led to substantial study to study inconsistencies in the classification of pericytes, which in turn confounds the interpretation of research on pericyte functions. Thus, a comprehensive characterization of pericyte subtypes and validation of appropriate identifying markers for these populations are crucial next steps for advancing our knowledge of pericyte functions and differentiation potential. Single cell sequencing offers tremendous promise in establishing global gene expression signatures and novel molecular identifiers of pericytes, as well as enabling the discovery of subtype, tissue and disease-specific patterns of pericyte gene expression. With this knowledge, it will be possible to evaluate the extent to which pericyte subpopulations are pre-programed with discrete and tissue-specific lineage potentials and to identify which pericyte subgroups have the most favorable characteristics for use in regenerative cell therapies.

Emerging evidence indicates that the regulation of metabolic pathways may offer the potential to manipulate the cell-state and fate decisions of pericytes, which could enable the development of novel strategies to control pericytes contributions to pathological conditions and their use in regenerative medicine. However, knowledge of pericyte metabolism is rudimentary to date and the overall understanding of how pericytes integrate signal transduction, metabolic programing and cell state/fate decisions currently lags far behind the state of knowledge of these relationships in other vascular and stem cells. Therefore, many fundamental questions remain to be answered. Considering the extent of pericyte heterogeneity *in vivo*, it will be important to determine if there is variability in metabolic programing that coincide with specific gene expression signatures. With the evolving advancements in metabolomics technologies, there is now unprecedented opportunity to map the metabolome of pericytes even to the single cell level and to identify the metabolic pathways that are integral to transitions in pericyte state and lineage commitment. Lastly, it is crucial to consider the impact of various pericyte isolation and culturing conditions on the signaling and metabolic pathways that regulate activation and differentiation potential, since this could greatly influence the reproducibility and efficacy of cell-based therapeutic treatments. Addressing these avenues of research not only will provide valuable insight into the contribution of specific metabolic pathways in shaping pericyte phenotype but it may also reveal new tools for the optimization of pericyte characteristics in pathological conditions and when used in cell therapies.

## Author Contributions

All authors listed have made a substantial, direct and intellectual contribution to the work, and approved it for publication.

## Conflict of Interest

The authors declare that the research was conducted in the absence of any commercial or financial relationships that could be construed as a potential conflict of interest.

## Footnotes

1 http://betsholtzlab.org/VascularSingleCells/database.html
